# Exploring the in-vitro evaluation of folic acid-modified gadolinium nanoparticles in HEK-293, HeLa, and KB cancer cells for targeted-MR-imaging agent candidate

**DOI:** 10.1038/s41598-026-38700-7

**Published:** 2026-08-07

**Authors:** Retna Putri Fauzia, Azmi Aulia Rahmani, Riezki Amalia, Ratna Dini Haryuni, Irkham Irkham, Husein H. Bahti, Santhy Wyantuti

**Affiliations:** 1 Department of Chemistry, Faculty of Mathematics and Natural Sciences, Universitas Padjadjaran, Jl. Raya Bandung-Sumedang Km. 21 Jatinangor, Sumedang, 45363 Indonesia; 2 Department of Pharmacology and Clinical Pharmacy, Faculty of Pharmacy, Universitas Padjadjaran, Jl. Raya Bandung-Sumedang Km. 21 Jatinangor, Sumedang, 45363 Indonesia; 3 Radiopharmaceuticals, and Biodosimetry Technology, Research Center for Radioisotopes, Research Organization for Nuclear Energy - National Research and Innovation Agency, KST BJ Habibie, Tangerang Selatan, 15314 Indonesia

**Keywords:** Contrast agent, Folic acid, Gadolinium nanoparticles, Magnetic resonance imaging, Nanoparticles internalization, Biotechnology, Cancer, Chemistry, Drug discovery, Nanoscience and technology

## Abstract

Folic acid (FA)-modified gadolinium nanoparticles are promising candidates for magnetic resonance imaging (MRI) contrast agents due to their high cancer specificity. Therefore, this study aims to modify nanoparticles with polyethylene glycol (PEG)-FA to enhance their biocompatibility and specificity toward cervical cancer target via folate receptor (FR)-mediated endocytosis. Spherical nanoparticles less than 20 nm were characterized by dynamic light scattering and transmission electron microscopy, with UV absorbance peaks at 282 and 362 nm confirming the successful attachment of FA. Additionally, the zeta potential at − 51.23 mV indicated good colloidal stability. Subsequently, an in vitro investigation was performed using three different FA-expressing cancer cell lines: HEK-293 (−), HeLa ( +), and KB (+ +), demonstrating that the cancer cells remained > 90% viable after 24 h incubation with the nanoparticles. There was enhanced FA-nanoparticle uptake in HeLa and KB, with minimal uptake in HEK-293 cells. In addition, FA pretreatment reduced uptake, confirming the receptor specificity. Compared to nanoparticles without modification, modified nanoparticles showed a significant increase in uptake in cancer cells expressing the FA receptor (*p* < 0.0001). These results support the potential of FA-modified gadolinium nanoparticles as a receptor-targeted contrast agent for further cervical cancer MR imaging applications.

**Supplementary Information:**

The online version contains supplementary material available at https://doi.org/10.1038/s41598-026-38700-7.

## Introduction

The high cancer mortality rate is a global challenge (according to WHO report)^1^, with cervical cancer ranked the fourth leading cause of cancer death in 2022^2^. Hence, timely diagnosis and effective treatments, such as radiotherapy and/or chemotherapy, are crucial to reduce these mortality rates^3–5^. Several imaging modalities have been utilized, including magnetic resonance imaging (MRI), ultrasonography (US), computed tomography (CT), positron emission tomography (PET), and single-photon emission computed tomography (SPECT)^6–10^. MRI is preferred primarily because it uses magnetic forces to acquire high-resolution soft-tissue images, with no ionizing radiation^11^. Contrast agents are commonly used to improve MRI specificity, so research is ongoing to develop less toxic MRI agents for better tissue differentiation.

The use of contrast agents enhances the relaxation properties of water protons and improves signal differentiation between normal and pathological tissues, thereby improving soft-tissue contrast^12^. Commonly used MRI contrast agents include paramagnetic ion complexes, such as gadolinium (Gd^3+^) and manganese (Mn^2+^), which function as T_1_ contrast agents^13–16^, as well as superparamagnetic iron oxide nanoparticles (SPIONs)^17–22^ as T_2_ contrast agents. T_1_-based contrast agents increase the relaxation rate (r_1_) and produce bright images by shortening the T_1_ relaxation rate of water protons. Meanwhile, T_2_-based contrast agents increase the relaxation rate (r_2_) and produce dark images by shortening the transverse relaxation time T_2_ of water protons^7,23–25^. Typically, Gd is used because it has seven unpaired electrons in the 4f. orbital (S = 7/2)^26–28^, making it highly effective in accelerating the longitudinal relaxation (T_1_) of water protons, thereby enhancing signal intensity in T_1_-weighted MRI scans^29,30^. Several clinically approved Gd-based agents include Magnevist (Gd-DTPA), Omniscan (Gd-DTPA-BMA), MultiHance (Gd-BOPTA), Dotarem (Gd-DOTA), Gadavist (Gd-BT-DO_3_A), and ProHance (Gd-HP-DO_3_A)^7^.

Although conventional Gd chelates are commonly used in MRI diagnostics, these materials have several limitations, including relatively low thermodynamic and kinetic stability. In addition, several studies have reported Gd deposition in the bone and brain of patients with normal renal function^31,32^ .Given these limitations, the development of MRI contrast compounds based on nanoparticle systems has become a major focus. Gd-based nanoparticles offer several advantages, such as lower toxicity, more controlled biodistribution, prolonged circulation time, and enhanced contrast efficiency, even at lower doses^33–36^. The important role of nanoparticles as MRI contrast-enhancing agents is to improve image contrast and to selectively accumulate in tumor cells, both of which can be achieved through nanoparticle surface functionalization. Nanoparticles are non-specifically accumulated in tumors via passive targeting, which involves enhanced permeability and retention (EPR)^37^. Passive targeting may not provide sufficient contrast in tumor-accumulated tissues, making active targeting with ligands essential to improve the specificity and efficacy of MRI contrast agents. By modifying nanoparticles’ surfaces with tumor-targeting ligands, active targeting enhances both accumulation and specificity, selectively binding to receptors overexpressed on tumor cell membranes^38–41^.

Functionalizing nanoparticles with targeting ligands that bind with high affinity to specific tumor cells is the most effective strategy to promote their attachment to cancer cells. Folic acid (FA) is a well-known cancer-targeting ligand due to its high binding affinity (Kd ≈ 10^−10^ M) to folate receptor (FR)^17,42^. Several studies have shown that the receptor is overexpressed in various human carcinomas, including breast, ovary, kidney, lung, and cervix^43^. FR is a glycosylphosphatidylinositol (GPI)-anchored membrane proteins that facilitate the transport of FA into cells through receptor-mediated endocytosis^44^. This targeting pathway has been widely exploited for site-specific delivery systems, including nanoparticle functionalization, to enhance specificity and intracellular uptake in FR-positive cancer cells^42^. Recent studies have demonstrated that FA-functionalized nanoparticles can enhance selective interactions with cancer cells, thereby improving the performance of nanomaterials in biomedical applications^17,18,45–47^.

Surface functionalization was verified using a range of physicochemical characterization techniques, including dynamic light scattering (DLS), Fourier transform infrared spectroscopy (FTIR), UV–visible spectrophotometry, transmission electron microscopy (TEM), and zeta potential measurements. A resazurin-based viability assay subsequently evaluated the biocompatibility of the resulting nanoparticles to assess their suitability for biomedical use. Cellular internalization was further examined by flow cytometry in three cell lines—HeLa, HEK-293, and KB—representing different levels of folate receptor expression. Through the approach, this study not only validates the effectiveness of the developed nanoparticle design but also offers new insights into their potential as more specific, stable, and safe MRI contrast agents for future biomedical applications.

## Result and discussion

### Synthesis and characterization of folic acid-modified gadolinium nanoparticles (FA-GdNPs)

**Fig. 1:**
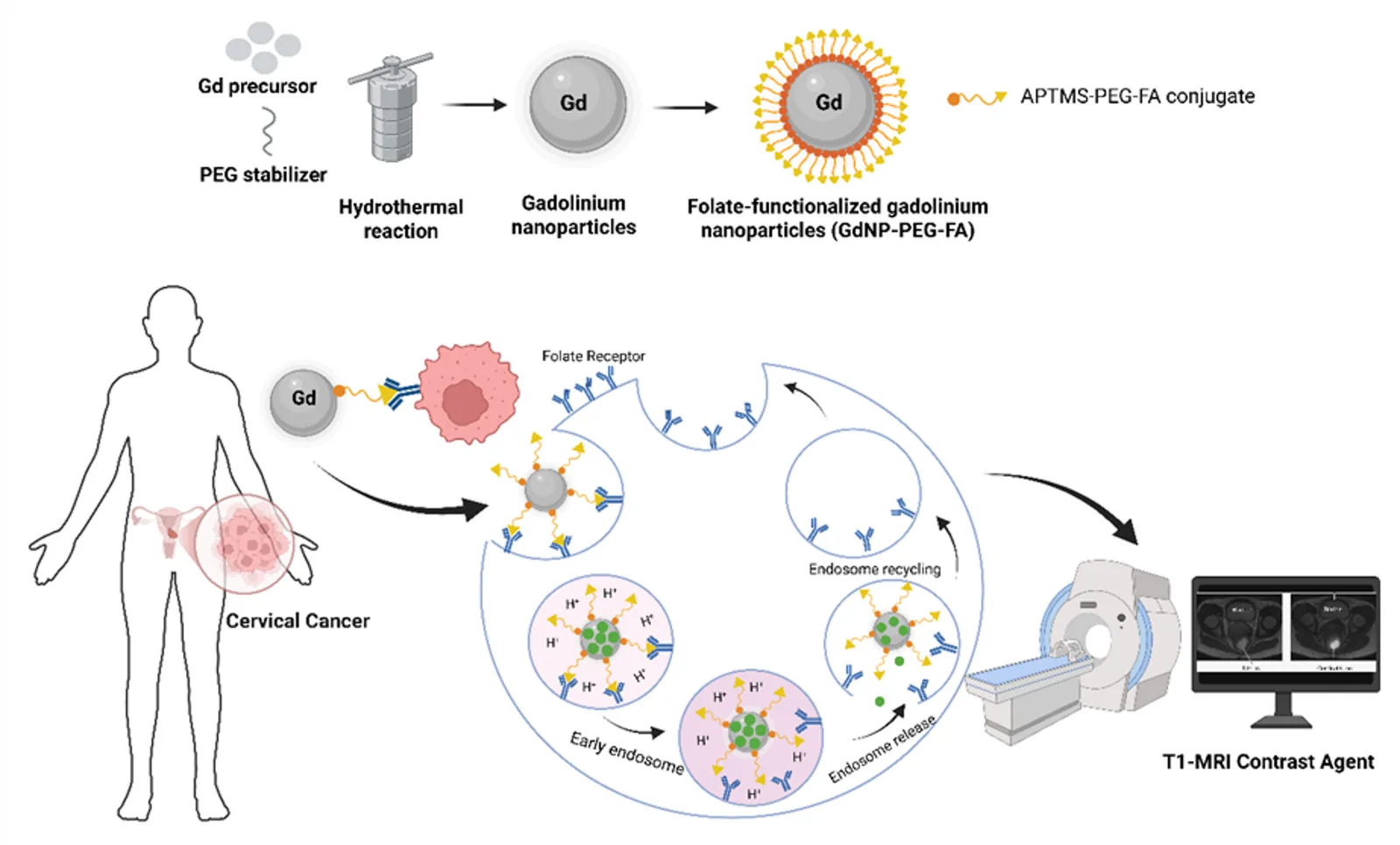
Schematic illustration of FA-GdNPs synthesis and FR–mediated endocytosis, enhancing tumor targeting and MRI sensitivity in cervical cancer detection.

Gd nanoparticles were synthesized using a hydrothermal, water-based method involving nucleation of Gd^3+^ precursors, followed by particle growth in solution^48^. PEG was incorporated as a stabilizing agent to prevent aggregation. The presence of PEG enhances colloidal stability because its hydrophilic properties and negatively charged carboxyl groups can interact with the positively charged Gd^3+^ ions on the nanoparticle surface. To improve active targeting, the nanoparticles were functionalized with FA, enabling specific recognition and binding to FR-positive cancer cells. This functionalization can significantly improve the specificity and precision, enabling active recognition and binding to FR-positive cancer cells, which are known to be overexpressed in several cancer types, including cervical cancer. In addition, PEGylation prolonged the systemic circulation time of the nanoparticles by minimizing immune recognition and uptake by the reticuloendothelial system (RES), thereby improving their overall performance as an MRI contrast agent. Uptake of FA-GdNPs by cancer cells primarily occurred via FR-mediated endocytosis, leading to greater intracellular accumulation and improved imaging sensitivity (Fig. 1).

PEG-FA was functionalized via an EDC/NHS-mediated coupling reaction. The carboxyl group of folic acid was first activated to form an NHS ester, which reacted with amine-terminated PEG to produce FA-PEG-COOH. This conjugate was then linked to APTMS, yielding FA-PEG-APTMS, which was used for subsequent surface functionalization of gadolinium nanoparticles via silane coupling.

**Fig. 2:**
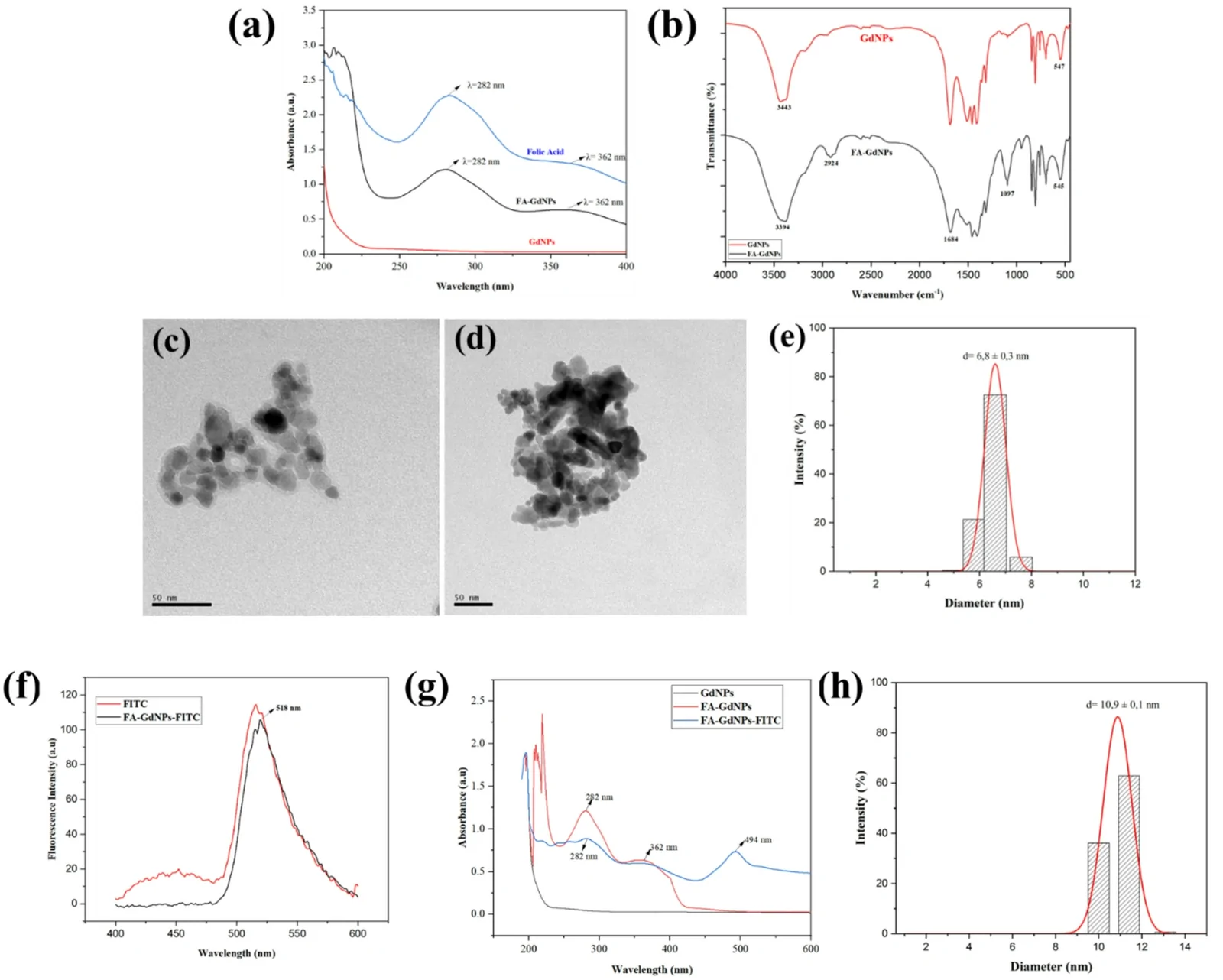
Characterization of FA-GdNPs: (**a**) UV–Vis and (**b**) FTIR spectra confirming successful FA conjugation; (**c**), (**d**) TEM images and (**e**) DLS analysis illustrating morphological properties and uniform size distribution.

Successful conjugation of PEG and FA onto GdNPs was confirmed by FTIR and UV spectroscopy. The FTIR spectra (Fig. 2b) provided clear evidence of the successful surface modification of GdNPs with PEG and FA. As shown in Fig. 1, the spectrum of FA-GdNPs exhibited a broad band at 3384 cm^−1^ corresponding to O–H stretching vibrations of hydroxyl groups on the surface of Gd_2_O₃, a peak at 2918.62 cm^−1^ attributed to C–H stretching of PEG, and a sharp peak at 1684.26 cm^−1^ corresponding to C = O stretching, indicating the presence of FA moieties. Additional peaks at 1097.55 cm^−1^ (C–O–C stretching) and 552.08 cm^−1^ (Gd–O vibration) further supported the successful incorporation of PEG and confirmed the structural integrity of the GdNPs core. Complementary evidence was provided by UV–Vis spectroscopy (Fig. 2a), which showed two distinct absorption peaks at 282 nm and 362 nm in the FA-GdNPs, corresponding to the π → π* and n → π* transitions of the aromatic and carbonyl groups in FA^16,49^ respectively. These peaks were absent in GdNPs, confirming the successful covalent attachment of FA to the nanoparticles’ surface. To verify successful surface functionalization, we quantified FA conjugation by UV spectroscopy, yielding a concentration of 0.0784 mM per mg of FA-GdNPs. This attachment is pivotal for nanoparticle performance by enabling selective recognition of FR-overexpressing cancer cells, such as HeLa and KB, and facilitating receptor-mediated endocytosis. This targeted approach is designed to maximize the accumulation of the contrast agent specifically at the tumor site, thereby improving diagnostic clarity.

DLS analysis (Fig. 2e and Table 1) showed that FA-GdNPs have an average hydrodynamic diameter of 6.8 nm with a uniform distribution. Morphological analysis using TEM in Figs. 2c and d shows that FA-GdNPs have a spherical shape with minor aggregation. This aggregation may result from interactions between surface functional groups on the nanoparticle. Despite the aggregation, the nanoparticles remain below 20 nm in size. Nanoparticle size strongly influences the behavior of contrast agents, including targeting efficiency, cellular uptake, and in vivo distribution. Smaller particles expose more Gd^3+^ on their surface, which enhances T1 relaxation and improves MRI signal intensity, while nanoparticles in the 5–10 nm range are better able to penetrate deeper tumor cells^50^. Although particles below 10 nm may undergo rapid renal clearance, this feature can be advantageous by minimizing long-term retention and toxicity in non-target organs, such as the liver, spleen, and lungs^40^. Given their optimized size, active FA-based targeting, and PEGylation, which supports longer circulation time, FA-GdNPs show strong potential as a nanomedicine platform for effective and safe MRI contrast agents.

**Table 1:** Characterization of FA-GdNPs.

Parameter	FA-GdNPs
Size distribution	6.8 nm
Zeta Potential	− 51.23 ± 0.2081 mV
Concentration of folic acid (of 1 mg FA-GdNPs)	0.0784 mM
Concentration of Gd (inductively coupled plasma optical emission spectrometer)	35.41 ± 0.24%
Concentration of free Gd (UV–Vis spectrophotometer)	2.38 ± 0.0046%

The colloidal stability of FA-GdNPs was assessed by their zeta potential as a key indicator of interparticle repulsion. The particles exhibited a mean value of − 51.23 mV (Table 1), suggesting a surface charge that prevents them from sticking to another^51^. This strong electrostatic repulsion is critical, as it prevents nanoparticles from aggregating in suspension, thereby maintaining colloid stability for further application. In contrast, bare GdNPs showed a positive ζ-potential of + 36.7 ± 0.80 mV^48^. The decrease in ζ-potential following functionalization could be attributed to the introduction of negatively charged hydroxyl and carboxyl groups from the polymer coating. Such highly negative ζ-potential values, in combination with small particle size, are advantageous for efficient cancer cell uptake through enhanced electrostatic interactions^52^.

Furthermore, 1 mg of FA-GdNPs contained a total Gd concentration of 35.41 ± 0.24%, as determined using inductively coupled plasma-optical emission spectrometry (ICP-OES) (Table 1). The free Gd(III) content was assessed using a UV–Vis spectrophotometer with xylenol orange as the indicator and was found to be only 2.3833 ± 0.0046%. Gadolinium-based contrast agents enhance MRI signal intensity through the paramagnetic properties of trivalent gadolinium (Gd^3^⁺), which has seven unpaired electrons that accelerate proton relaxation and shorten the longitudinal relaxation time (T_1_)^53^. A higher total Gd content, therefore, increases the number of active paramagnetic centers, resulting in stronger T_1_-weighted image contrast. Nevertheless, strict control of free Gd(III) ions is essential because of their known toxicity, particularly their ability to interfere with calcium-dependent biological pathways and to accumulate in tissues, which poses elevated risks for individuals with impaired renal function^32^. Crucially, the minimal release of free Gd(III) ions indicates that it remained securely trapped within the FA-GdNPs, significantly reducing potential toxicity without compromising the high relaxivity required for effective imaging.

Fluorescein isothiocyanate (FITC) was conjugated to the FA-GdNPs to facilitate internalization studies as a fluorophore marker. The emission spectrum in Fig. 2f reveals a distinct peak at 518 nm, characteristic of FITC, with a concentration of 0.017 ± 0.0001 mmol per mg of FA-GdNPs. The UV–Vis spectra in Fig. 2g provided additional evidence: the peaks at 480 and 500 nm correspond directly to the π → π* transitions of the FITC-conjugated system. The increased absorbance around 280 nm confirms the presence of FA. DLS analysis in Fig. 2h shows an increased hydrodynamic size to 10.9 nm. The nanoparticles maintained the monodisperse distribution necessary for intracellular tracking.

### Stability studies

**Fig. 3:**
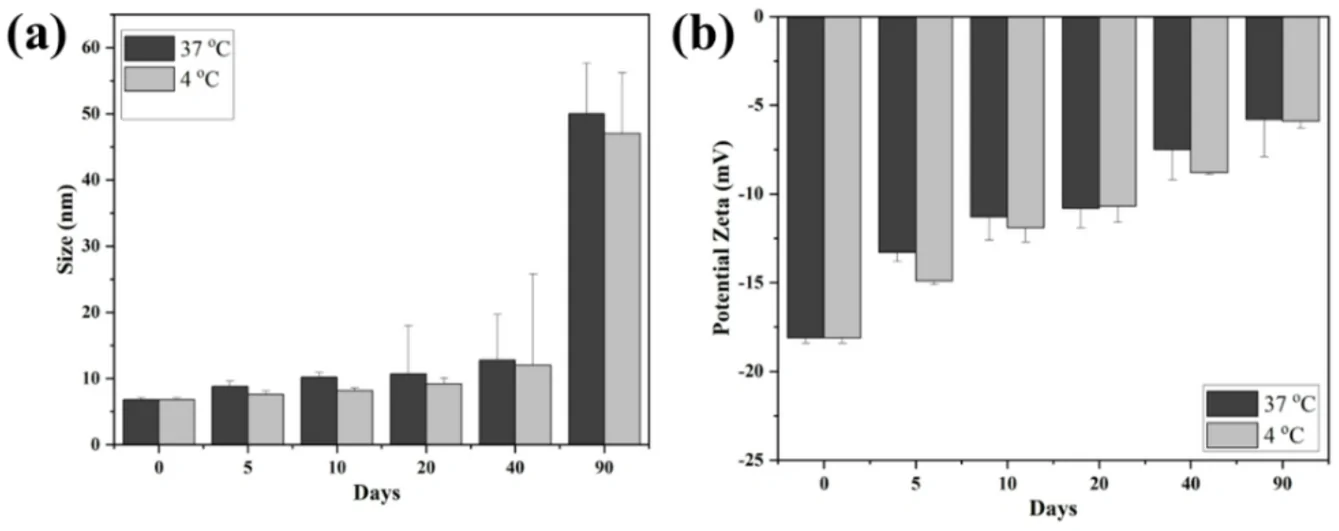
(**a**) Size stability and (**b**) Zeta potential stability of FA-GdNPs nanoparticles stored for 90 days at 4 °C and 37 °C.

Product stability is an important factor in the development and application of nanoparticles as new candidates for MRI contrast agents, given their effectiveness and safety^54^. Maintaining the physicochemical stability of nanoparticles during storage is crucial to preserving their particle size and surface charge characteristics. In this study, FA-GdNPs demonstrated excellent colloidal stability over 20 days when stored in PBS (pH 7,4) at both 4 °C and 37 °C. As shown in Tables S4 and S5, the hydrodynamic diameter is consistently stable under 10 nm, while the zeta potential remains above − 10 mV (Fig. 3). The observed fluctuations in surface charge are attributed to the ionic environment of the physiological buffer. Although the deprotonated carboxylate groups of FA provide a baseline negative charge, the abundance of Na^+^, Cl^-^, H_2_PO_4_^-^, and HPO_4_^2-^ ions in PBS leads to electrostatic screening^55,56^. By interacting with the surface -COO- groups, these counterions compress the electric double layer^57,58^. This phenomenon naturally reduces the measured surface charge without compromising the overall stability of the nanoparticle system. Previous studies have reported a decrease in zeta potential from − 59 mV in water to − 29 mV after polymer nanoparticles were dispersed in PBS^59^. These results demonstrate the critical role of PBS ions in modulating nanoparticle surface charge and colloidal stability. Preservation of nanoparticle size in the sub − 10 nm range was favorable, as longitudinal relaxivity (r₁) has been shown to increase significantly at particle sizes below 15 nm.^30,60–62^. Therefore, maintaining the colloidal stability of FA-GdNPs under physiological conditions supported their potential efficacy as high-performance T₁ MRI contrast agents.

### Viability

**Fig. 4:**
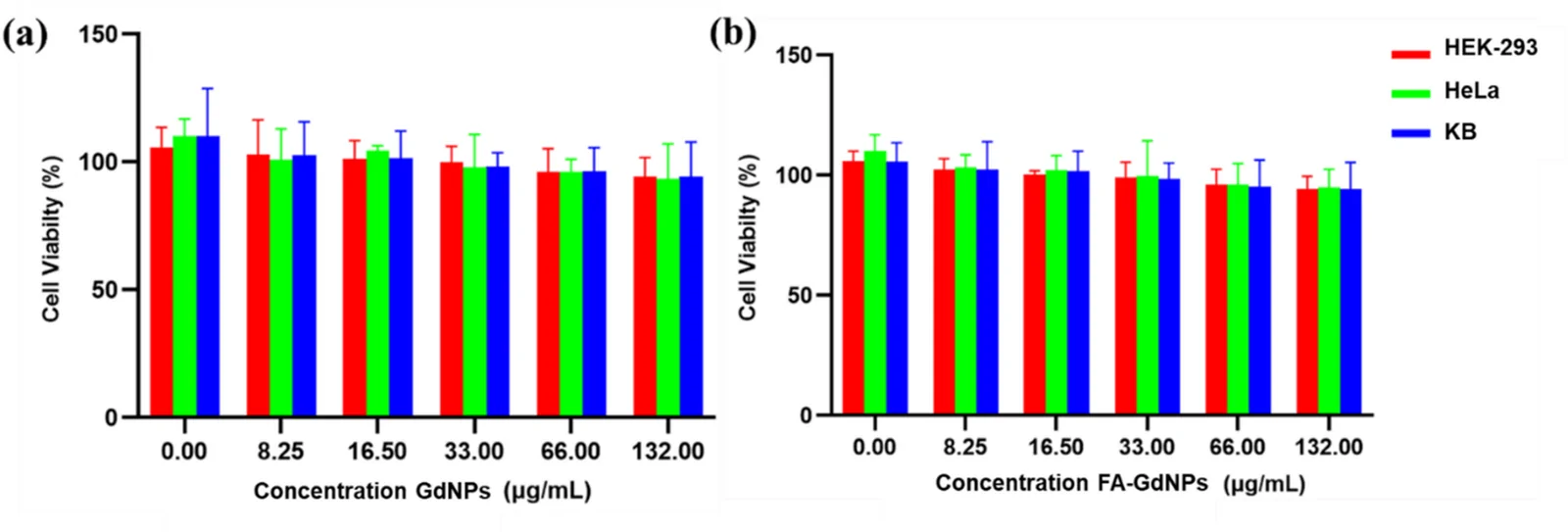
In vitro cytotoxicity of (**a**) GdNPs and (**b**) FA-GdNPs against HEK-293 (red), HeLa (green), and KB (blue) cell lines. Viability was assessed using the Resazurin assay following 24 h of treatment at concentrations ranging from 0.0 to 132.0 µg/mL (n = 5).

Cell viability was evaluated using the resazurin reduction assay, which measured the capacity of metabolically active cells to convert the non-fluorescent dye resazurin into its fluorescent product, resorufin. In this study, three cell lines with different levels of FR expression were selected, namely HEK-293, a human embryonic kidney cell line with low FR expression^63–65^, HeLa, a cervical cancer cell line with moderate FR expression^66,67^, and CCL-17/KB, a HeLa-derived cell line characterized by high FR expression, serving as the high-expression model^67–70^. These models were used to assess not only the cytotoxicity profile of the nanoparticles but also their potential selectivity toward FR-expressing cancer cells. Cells were incubated for 24, 48, and 72 h with increasing concentrations of nanoparticles (8.25–132 µg/mL), followed by resazurin treatment and fluorescence quantification. Cell viability at 24 h of incubation in Fig. 4 shows that it remains high (> 90%) at all concentrations tested, with no significant differences (GdNPs vs FA-GdNPs) in all cell lines. After 48 h of incubation, cell viability remained above 70%, indicating no change in cellular metabolic activity (Figure S3). Interestingly, decreased cell viability below 70% after 72 h across all cell models (Figure S4) suggests that decreased metabolic activity is likely a consequence of extended exposure rather than a toxic response to the nanoparticle concentration. Given these findings, we concluded that a 24-h incubation is more suitable for evaluating the cytocompatibility of nanoparticles. The findings indicate that functionalization with PEG and FA did not induce additional cytotoxicity, in line with previous reports demonstrating the good biocompatibility of PEGylated and folate-modified^17,49^. Furthermore, the low cytotoxicity toward normal cells (HEK-293) and FR-positive cancer cells (HeLa and KB) further supports the potential of FA-GdNPs for in vitro research.

### Internalization

The study of cellular internalization is key to nanoparticles intended for use as MRI contrast agents, as it can influence targeting specificity, intracellular distribution, retention time, and ultimately imaging quality. Flow cytometry was used to measure the cellular uptake of GdNPs and FA-GdNPs, focusing on the role of FR density. Three distinct cell lines were selected to represent different expression levels: the FR-negative HEK-293 (−), FR-positive HeLa ( +), and the high-expressing KB (+ +) models.

**Fig. 5:**
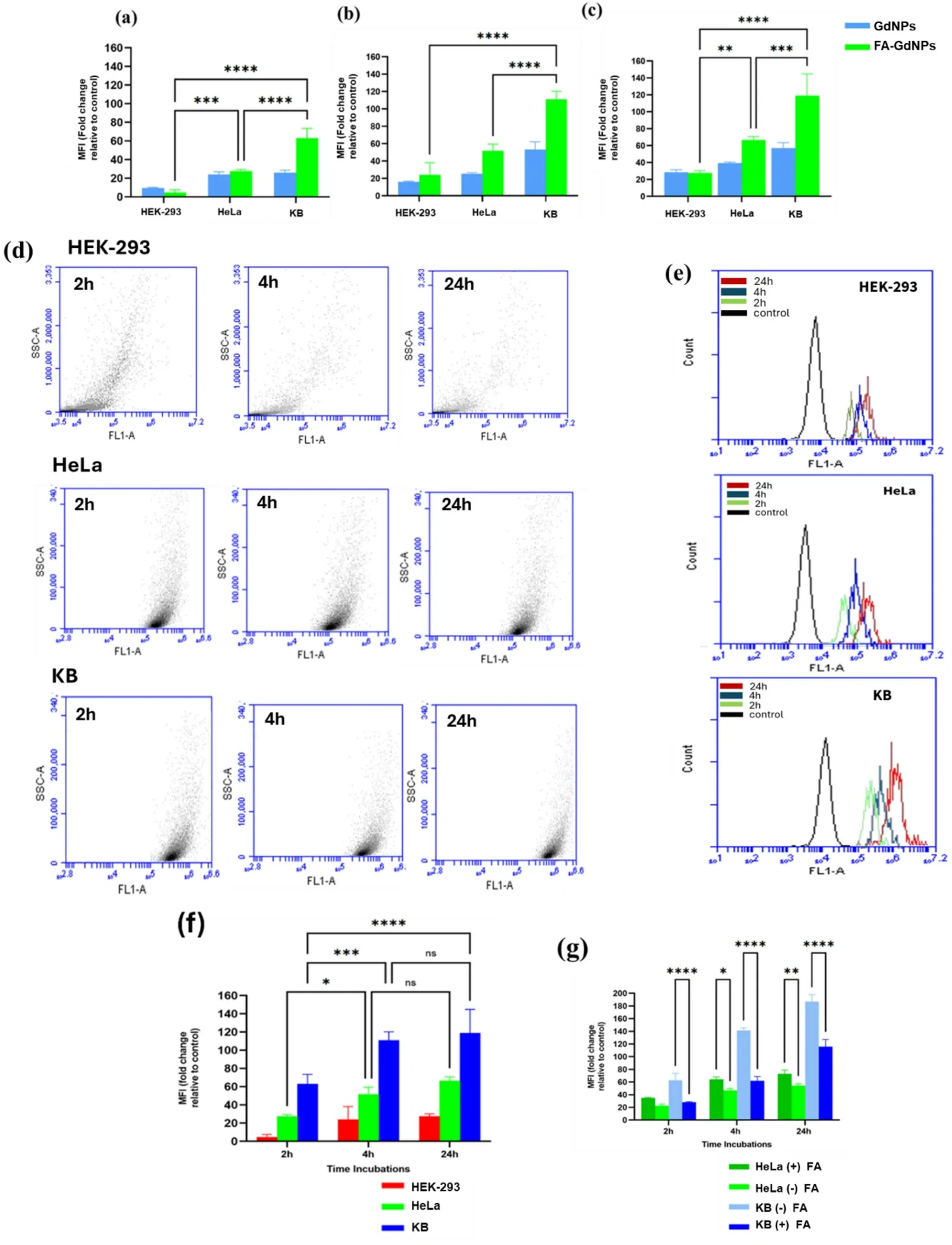
Cellular uptake profiles of GdNPs and FA-GdNPs. Time-dependent internalization in three cell lines (HEK-293, HeLa, and CCL-17) analyzed via flow cytometry (**a**)–(**c**). (**d**) Scatter plots (SSC-A vs. FL1-A) and (**e**) histograms show the shift in fluorescence intensity following nanoparticle treatment. (**f**) MFI fold changes relative to control across all time points. (**g**) FA-GdNPs uptake specificity in HeLa and CCL-17 cells evaluated by FA-mediated inhibition. Quantitative results are shown as mean ± SD (n = 3) and analyzed using two-way ANOVA followed by Tukey’s test for multiple comparisons (*p* < 0.05 to ***p < 0.0001).

As illustrated in Fig. 5a–c, both GdNPs and FA-GdNPs exhibit minimal internalization in HEK-293 cells, with average fluorescence intensity (MFI) values below 35 and no significant difference between them. This indicates that nonspecific internalization can be ignored, and, more importantly, that PEGylation and FA conjugation do not increase background uptake in FR-negative cells. This is crucial, as unwanted nonspecific interactions are a significant concern in nanoparticle delivery systems. In contrast, nanoparticle internalization was significantly increased in FR-positive cells. HeLa ( +) cells showed significantly higher uptake of FA-GdNPs after 24 h compared to non-functionalized GdNPs (**p < 0.01). A more substantial effect was detected in KB (+ +) cells, which express high levels of FR, as reflected by significantly increased MFI values exceeding 120 (****p < 0.0001). The apparent dependence between uptake efficiency and FR expression levels provides strong evidence that FA functionalization effectively promotes receptor-specific internalization, consistent with previous studies on folate-modified magnetic nanoparticles^17,18^.

Time-dependent changes in fluorescence intensity were first apparent in the representative dot plots (Fig. 5d) and histogram overlays (Fig. 5e), which showed a gradual rightward shift with increasing incubation time. This trend was subsequently confirmed by quantitative analysis (Fig. 5f). In KB cells, MFI increased substantially between 2 h (34.9 ± 4.8) and 4 h (111.7 ± 6.2), whereas only a minor increase was observed at 24 h (119.1 ± 5.7). Compared to KB cells, HeLa cells showed a slower uptake, with MFI values rising more modestly from 29.1 ± 3.5 at 2 h to 66.0 ± 3.9 at 24 h. In contrast, HEK-293 cells showed minimal variation in fluorescence intensity throughout the incubation period, indicating limited nanoparticle internalization. The early and pronounced increase in MFI observed in KB cells indicates that nanoparticle uptake predominates during the initial hours of incubation. The limited rise in MFI beyond 4 h suggests that uptake becomes constrained over time, likely due to the limited availability of FR binding sites. The plateau phase observed in FR-positive cells can be explained by receptor saturation and recycling dynamics, as reported in studies of FR trafficking^66,69,71^. The slower uptake kinetics observed in HeLa cells are consistent with their lower FR-expression relative to KB cells, resulting in a reduced internalization rate.

Free FA pretreatment was performed to verify the involvement of FR-mediated endocytosis through competitive inhibition of nanoparticle internalization. In Fig. 5g, without free folic acid treatment, both HeLa and KB cells showed time-dependent internalization of FA-GdNPs, with KB cells reaching the highest MFI (~ 180 at 24 h). A significant decrease in HeLa cells was detected at 4 h (*p < 0.05) and 24 h (**p < 0.01), while KB cells showed more potent inhibition at both time points (4 h (**p < 0.01) and 24 h (****p < 0.0001)). These results clearly indicate that FA-GdNPs uptake is primarily mediated by the FR pathway rather than by nonspecific endocytosis.

Based on the results of a two-way ANOVA test combined with a Tukey post-hoc analysis (Table S6), cell type, incubation duration, and folic acid pretreatment were found to significantly affect nanoparticle internalization (*p* < 0.005–0.0001). In general, the efficiency of FA-GdNPs internalization showed a pattern of KB > HeLa > HEK-293, which was consistent with the differences in folate receptor expression levels. These results confirm two main advantages of FA-GdNPs, namely (i) the ability to accumulate rapidly through a receptor-dependent mechanism in tumor cells with high FR expression, and (ii) low background uptake in non-target cells. These characteristics support the potential of FA-GdNPs as a targeted MRI contrast agent with improved tumor selectivity and enhanced imaging performance.

## Conclusion

In summary, gadolinium nanoparticles were successfully functionalized with PEG-FA, resulting in FA-GdNPs with confirmed surface modification. The UV–Vis spectra of FA-GdNPs show absorption bands at around 282 and 362, which are characteristic of folic acid and indicate effective FA conjugation onto the nanoparticles. FTIR analysis, where absorption peaks near 2918 and 1864 cm^−1^ correspond to PEG and FA functional groups, respectively, confirming the presence of FA on the nanoparticle surface. TEM images reveal that the nanoparticles remain predominantly spherical, with sizes below 20 nm, although a higher degree of aggregation is observed after surface functionalization. DLS analysis further confirms the stability and dispersibility of FA-GdNPs, with a hydrodynamic diameter of approximately 6.8 nm, maintaining good size uniformity for up to 20 days. Viability-resazurin assays using different cancer expression models, HEK-293 (−), HeLa ( +), and KB (+ +), show no significant difference in cell viability upon FA-attachment onto nanoparticles, suggesting that the modification does not increase cellular toxicity in cancer targets. Subsequently, flow cytometry analysis demonstrates enhanced uptake of FA-functionalized nanoparticles in FR–overexpressing cells (HeLa and KB) compared to normal cells (HEK-293), with further confirmation following prior incubation with the free FA molecule. In conclusion, FA-GdNPs are a promising candidate for a targeted MR-imaging agent for cervical cancer diagnosis. Nevertheless, in vivo demonstration will also play a critical role in proving designed concepts for further biomedical applications.

## Methods

### Materials

The materials used in this study were predominantly obtained from Sigma-Aldrich, including 1-ethyl-3-(3-dimethylaminopropyl) carbodiimide hydrochloride (EDC), (3-aminopropyl)-trimethoxysilane (APTMS, 97%), acetic acid (CH₃COOH), acetone, amino-PEG-acid (H_2_N-PEG-COOH), deionized water, dimethyl sulfoxide (DMSO), Dulbecco’s Modified Eagle Medium (DMEM), Eagle’s Minimum Essential Medium (EMEM), and ethylenediaminetetraacetic acid tetrasodium salt dihydrate (EDTA). Others included Fetal Bovine Serum (FBS), fluorescein isothiocyanate (FITC), FA, gadolinium oxide (Gd_2_O₃, 99.9%), N-hydroxysuccinimide (NHS), paraformaldehyde (PFA), phosphate-buffered saline (PBS), polyethylene glycol 6000 (PEG-6000), potassium bromide (KBr), resazurin, sodium acetate (CH₃COONa), trypsin–EDTA, and xylenol orange. The nitric acid (HNO₃, 65%) used in this study was obtained from Merck.

### Synthesis of gadolinium nanoparticles

Gd nanoparticles were synthesized using the hydrothermal method. Solid Gd_2_O₃ (2.455 g) was added to 5 mL of 65% HNO₃ on a hot plate, followed by the addition of 100 mL of Milli-Q water, and stirred with a magnetic stirrer until fully dissolved. PEG-6000 (75 g) was heated using the hot plate, and the Gd_2_O₃ solution was added to the melted PEG-6000. The resulting mixture was transferred to an autoclave and heated at 180 °C for 7 h, while the colloid was centrifuged 3 times and washed several times with acetone. The resulting solid powder was dried in an oven at 60 °C for 24 hours^48^.

### Preparation of silanes conjugated polyethylene glycol folic acid (APTMS-PEG-FA)

FA (44.1 mg, 0.1 mmol) was dissolved in 1 mL of anhydrous dimethyl sulfoxide (DMSO), and 1-ethyl-3-(3-dimethylaminopropyl) carbodiimide hydrochloride (EDC, 38.2 mg, 0.2 mmol) was added. The mixture was stirred for 3 h, then N-hydroxy succinimide (NHS, 11.5 mg, 0.1 mmol) was added and stirred for 24 h in the dark. The formation of FA-NHS was confirmed by UV spectroscopy.

A total of 10.6 mg of H_2_N-PEG-COOH was dissolved in 0.01 mmol of NHS-FA and stirred at room temperature for 24 h. The solution was centrifuged (10,000 rpm, 5 min), and the supernatant was dialyzed for 2 days against H_2_O. The resulting FA-PEG-COOH solution was freeze-dried and dissolved in 500 μL DMSO. Subsequently, 2.50 μL (0.01 mmol) of (3-Aminopropyl) trimethoxy silane (APTMS) was added. The mixture was stirred at room temperature for 24 h, and the APTMS-PEG-FA conjugate was analyzed using UV–VIS spectroscopy^49^.

### Preparation of folic acid functionalized gadolinium nanoparticles (FA-GdNPs)

Approximately 10 mg of Gd nanoparticles were dissolved in 5 mL of H_2_O and 5 mL of ethanol. The mixture was sonicated for 10 min using a sonicator bath. Subsequently, APTMS-PEG-FA solution was added and stirred for 24 h at room temperature. The mixture was then centrifuged and washed three times with ethanol, then dried under vacuum overnight.

### Labelling of folic acid functionalized gadolinium nanoparticles with FITC

FITC (0.025 mmol; 9.69 mg) was dissolved in 500 µL of ethanol and stirred at room temperature under dark conditions until an orange solution was obtained. Then, 0.01 mmol of APTMS was added, and the mixture was stirred for 24 h to form APTMS-FITC. The obtained APTMS-FITC was conjugated to 10 mg of FA-functionalized Gd nanoparticles dispersed in 5 mL of ethanol, and the mixture was sonicated for 10 min. The mixture was stirred at room temperature in the dark for 24 h, then centrifuged, washed 3 times with ethanol, and dried. The final product was characterized using UV–Vis and fluorescence spectrophotometers.

### Stability assessment

The stability of the FA-GdNPs was evaluated in PBS at pH 7.4 at 4 °C and 37 °C. Particle size and surface charge were evaluated using DLS and zeta potential on days 0, 5, 10, 20, 40, and 90.

### Cell culture

The cell lines used were HEK-293 (human embryonic kidney), HeLa (human cervical adenocarcinoma), and KB (human colorectal carcinoma). HEK-293 and HeLa cell lines were obtained from European Collection of Authenticated Cell Cultures (ECAAC), UK, while KB cell lines were obtained from American Type Culture Collection (ATCC), USA. All cell lines were maintained as adherent monolayers in T-25 tissue culture flasks under standard incubation conditions at 37 °C in a humidified atmosphere containing 5% CO_2_. HeLa and HEK-293 cells were cultured in Dulbecco’s Modified Eagle Medium (DMEM), while KB cells were cultured in Eagle’s Minimum Essential Medium (EMEM). Each culture medium was supplemented with 10% (v/v) Fetal Bovine Serum (FBS) and 1% (v/v) penicillin–streptomycin solution (10 mg/mL). Cells were routinely sub-cultured using a Trypsin–EDTA solution and utilized for experiments upon reaching 80–90% confluency. Before treatment, the cells were incubated in fresh complete medium for 24 h.

### Cell viability assay

HEK-293, HeLa, and KB cell lines were seeded into 96-well plates at a density of 0.6 × 10^4^ cells per well and incubated for 24 h at 37 °C in a humidified atmosphere with 5% CO_2_ until they reached approximately 80–90% confluency. After incubation, the medium in each well was replaced with fresh medium containing GdNPs and FA-GdNPs at varying concentrations (0–132 µg/mL). Subsequently, 100 µL of a resazurin solution (prepared by mixing resazurin dye with culture medium at a 1:9 ratio) was added to each well, and the cells were incubated for an additional 4 h at 37 °C with 5% CO_2_. Absorbance was measured at 570 nm and 600 nm using a microplate reader (n = 5)^72,73^.

### Internalization study

HEK-293, HeLa, and KB cells were seeded at 1.5 × 10^5^ cells/well in 6-well plates and incubated for 24 h at 37 °C with 5% CO_2_. The medium was then replaced with fresh medium containing FITC-labeled GdNPs or FA-GdNPs at 100 µg/mL, followed by incubation for 2, 4, and 24 h. After treatment, the medium was removed, and cells were washed twice with cold PBS (4 °C), detached using 0.05% trypsin–EDTA, and neutralized with cold complete medium. The cell suspension was transferred to 15 mL tubes, centrifuged at 2000 rpm for 7 min, and the supernatant was discarded. Cells were then fixed with 4% paraformaldehyde for 15 min and washed with cold PBS. After centrifugation, the supernatant was discarded, and the cell pellet was resuspended in 500 µL of 1 × PBS for flow cytometry. Samples were analyzed using the FITC channel with approximately 10,000 gated events per sample. Unlabeled cells served as negative controls^45^. Flow cytometric data were acquired using the BD Accuri C6 software (version 1.0.264.21) available at https://www.bdbiosciences.com.

### Statistical analysis

Statistical analysis was performed using one-way or two-way ANOVA with Tukey’s post hoc test (GraphPad Prism 10.5.0). Significance was set at *p* < 0.05, and data are presented as mean ± SD from 3 independent experiments (five replicates each).

## Supplementary Information

Below is the link to the electronic supplementary material.

Supplementary Material 1

## Data Availability

The datasets used and/or analyzed during the current study are available from the corresponding author on reasonable request.
