# Correction: Exploring the in-vitro evaluation of folic acid-modified gadolinium nanoparticles in HEK-293, HeLa, and KB cancer cells for targeted-MR-imaging agent candidate

**DOI:** 10.1038/s41598-026-70205-1

**Published:** 2026-09-07

**Authors:** Retna Putri Fauzia, Azmi Aulia Rahmani, Riezki Amalia, Ratna Dini Haryuni, Irkham Irkham, Husein H. Bahti, Santhy Wyantuti

**Affiliations:** 1 Department of Chemistry, Faculty of Mathematics and Natural Sciences, Universitas Padjadjaran, Jl. Raya Bandung-Sumedang Km. 21 Jatinangor, Sumedang, 45363 Indonesia; 2 Department of Pharmacology and Clinical Pharmacy, Faculty of Pharmacy, Universitas Padjadjaran, Jl. Raya Bandung-Sumedang Km. 21 Jatinangor, Sumedang, 45363 Indonesia; 3 Radiopharmaceuticals, and Biodosimetry Technology, Research Center for Radioisotopes, Research Organization for Nuclear Energy - National Research and Innovation Agency, KST BJ Habibie, Tangerang Selatan, 15314 Indonesia

Correction to: *Scientific Reports* https://doi.org/10.1038/s41598-026-38700-7, published online 07 August 2026

The original version of this Article contained an error in the spelling of the author Riezki Amalia which was incorrectly given as Riezki Amalia Putri.

The original Article has been corrected.

