# Supplementary material for: Exploring the in-vitro evaluation of folic acid-modified gadolinium nanoparticles in HEK-293, HeLa, and KB cancer cells for targeted-MR-imaging agent candidate
Source: Sci Rep. 2026 Aug 7;16:24462. doi: 10.1038/s41598-026-38700-7 (PMC13451495; doi:10.1038/s41598-026-38700-7)
Supplement: Supplementary file 1 [file 41598_2026_38700_MOESM1_ESM.docx]

**Supplementary Material**

**Exploring the In-Vitro Evaluation of Folic Acid-modified Gadolinium Nanoparticles in HEK-293, HeLa, and KB Cancer Cells for Targeted-MR-Imaging Agent Candidate**

*Retna Putri Fauzia^1*^, Azmi Aulia Rahmani^1^, Riezki Amalia Putri^2^, Ratna Dini Haryuni^3^, Irkham Irkham^1^, Husein H. Bahti^1^, and Santhy Wyantuti^1^*

^1^Department of Chemistry, Faculty of Mathematics and Natural Sciences, Universitas Padjadjaran, Jl. Raya Bandung-Sumedang Km. 21 Jatinangor, Sumedang 45363, Indonesia

^2^A Department of Pharmacology and Clinical Pharmacy, Faculty of Pharmacy, Universitas Padjadjaran, Jl. Raya Bandung-Sumedang Km. 21 Jatinangor, Sumedang 45363, Indonesia

^3^Research Center for Radioisotopes, Radiopharmaceuticals, and Biodosimetry Technology, Research Organization for Nuclear Energy - National Research and Innovation Agency, KST BJ Habibie, Tangerang Selatan 15314, Indonesia

*Corresponding author: [retna.fauzia@unpad.ac.id](mailto:retna.fauzia@unpad.ac.id). Phone +6281912444000

**Figure S1.** Curva Calibration of Gd Solution

**Figure S2.** UV-VIS Spectra of Xylanol Orange (XO, black) and FA-GdNPs (red)

**Figure S3.** In vitro cytotoxicity of (A) GdNPs and (B) FA-GdNPs against HEK-293 (red), HeLa (green), and KB (blue) cell lines. Viability was assessed using the Resazurin assay following 48 hours of treatment at concentrations ranging from 0.0 to 132.0 µg/mL (n = 5).

**Figure S4.** In vitro cytotoxicity of (A) GdNPs and (B) FA-GdNPs against HEK-293 (red), HeLa (green), and KB (blue) cell lines. Viability was assessed using the Resazurin assay following 72 hours of treatment at concentrations ranging from 0.0 to 132.0 µg/mL (n = 5).

**Tabel S1.** Comparasion of Important FTIR Functional Groups of GdNPs and FA-GdNPs

| **Sample** | **Wavenumber (cm⁻¹)** | **Functional Group Assignment** | **Remark** |
| --- | --- | --- | --- |
| **GdNP** | 3443 | O–H stretching (hydroxyl, adsorbed water) | Broad band typical for nanoparticles |
|  | 547 | Gd–O stretching | Characteristic Gd–O bond |
| **FA-GdNP** | 3324 | N–H stretching (folic acid) | New band, indicating FA presence |
|  | 2924 | C–H stretching (folic acid) | Characteristic of FA |
|  | 1644 | C=O stretching (amide) | Confirmation of amide/FA bonding |
|  | 1097 | C–N stretching (folic acid) | Presence of amine group from FA |

**Tabel S2.** Zeta Potential Data

| **Replicate** | **T**  **(^0^C)** | **ZP**  **(mV)** | **Mob**  **(µmcm/Vs)** | **Cond**  **(mS/cm)** |
| --- | --- | --- | --- | --- |
| 1 | 25.0 | -51.0 | -0.000398 | 0.128 |
| 2 | 25.0 | -51.4 | -0.000395 | 0.128 |
| 3 | 25.0 | -51.3 | -0.000397 | 0.128 |
| **Average** | | -51.23 |  |  |
| **Standard deviation** | | 0,2081 |  |  |

**Run 1	Run 2**

**Run 3**

**Tabel S3.** Practical concentration and total gadolinium percentage of FA-GdNP obtained by ICP measurement

| **Replicate** | **Intensity** | **Practical Concentration (mg/L)** | **% Total Gd** |
| --- | --- | --- | --- |
| 1 | 1,044,953 | 35.1297 | 35.1297 |
| 2 | 1,057,134 | 35.5402 | 35.5402 |
| 3 | 1,058,189 | 35.5758 | 35.5758 |
| **Average** |  | **35.4152** |  |
| **Standard deviation** |  | **0.2479** |  |

**Table S4**. Size Stability of GdNP-PEG-FA nanoparticle for 90 days at storage temperatures of (a) 4°C and (b) 37°C

| Storage time  (day) | Average Hydrodynamic Diameter of GdNP-PEG-FA (nm) | |
| --- | --- | --- |
|  | 4°C | 37°C |
| 0 | 6,8 ± 0,3 | 6,8 ± 0,3 |
| 5 | 7,6 ± 0,5 | 8,8 ± 0,8 |
| 10 | 8,2 ± 0,4 | 10,2 ± 0,7 |
| 20 | 9,2 ± 0,9 | 10,7 ± 7,3 |
| 40 | 12,0 ± 13,8 | 12,8 ± 6,9 |
| 90 | 12,8 ±47,92 | 50,0 ± 7,6 |

**Table S5.** Charge Stability of GdNP-PEG-FA nanoparticle for 90 days at storage temperatures of (a) 4°C and (b) 37°C

| Storage time  (day) | Average Potential Zeta GdNP-PEG-FA (mV) | |
| --- | --- | --- |
|  | 4°C | 37°C |
| 0 | -18,1 ± 0,3 | -18,1 ± 0,3 |
| 5 | -14,9 ± 0,2 | -13,3 ± 0,5 |
| 10 | -11,9 ± 0,8 | -11,3 ± 1,3 |
| 20 | -10,7 ± 0,9 | -10,8 ± 1,1 |
| 40 | -8,8 ± 0,1 | -7,5 ± 1,7 |
| 90 | -5,9 ± 0,4 | -5,8 ± 2,1 |

**Tabel S6.** ANOVA and Tukey Analysis of Nanoparticle Internalization

| **Factor** | **P-value** | **Tukey Post-hoc Result** |
| --- | --- | --- |
| GdNPs vs FA-GdNPs (Cell type effect) | p < 0.005 | GdNP: minor differences among HEK-293, HeLa, CCL-17  GdNP-PEG-FA: CCL-17 > HeLa > HEK-293 |
| FA-GdNPs (Effect of incubation time: 2, 4, 24 h) | p < 0.0001 | 2 h: HEK-293 vs HeLa p=0.0085; HEK-293 vs CCL-17 p=0.0335; HeLa vs CCL-17 ns (p=0.7941)  4 h: HEK-293 vs HeLa p=0.0186; other ns  24 h: All comparisons significant (p<0.001); CCL-17 > HeLa > HEK-293 |
| GdNP-PEG-FA (Effect of folic acid pretreatment + incubation time) | p < 0.0001 | 2–4 h: no significant differences among groups  24 h: CCL-17 (+FA and -FA) shows highest internalization; significant increase from 2 → 24 h. HeLa (+/-FA) shows slower internalization, not always significant. |
